# Supplementary material for: Carbon fiber reinforced composite material layup design optimization method and its application in automobile battery box
Source: PLoS One. 2025 Dec 29;20(12):e0322729. doi: 10.1371/journal.pone.0322729 (PMC12747345; doi:10.1371/journal.pone.0322729)
Supplement: Attachment — Submitted filename: Response to Reviewers.docx [file pone.0322729.s005.docx]

**Reviewer #1:**

**Dear reviewer:**

**We are very grateful for your professional review of our article. As you are concerned, there are several issues that need to be addressed. According to your suggestions, we have made a lot of revisions to the original manuscript, and the detailed revisions are as follows:**

1- The introduction provides good context but needs a clearer distinction of how this work differs from previous studies.

By combing through the above literature, the researchers have studied and analyzed the performance of automotive battery box assemblies from different angles, including structural optimization, high-strength and lightweight materials, and optimization algorithms, but have ignored manufacturing costs and universality. The lightweight design of the automotive bat-tery box assembly requires comprehensive consideration of factors such as safety, structural optimization, material application and cost control.

In terms of safety, the design and manufacture of the automotive battery box assembly requires comprehensive consideration of multiple factors to ensure the safe driving of the vehicle under various operating conditions. In terms of structural optimization, factors such as the ply angle, shape, number of layers and sequence of carbon fiber composite materials have an important influence on structural performance and strength. At the same time, facing the problem of multi-variable optimization, the convergence and optimization efficiency of the optimization algorithm need to be improved, and the quantitative ranking of the optimized compromise solutions will help to comprehensively balance the performance requirements of all parties. In terms of cost control, it is difficult for an automotive battery box assembly made of a single lightweight material to have both performance and cost advantages. In response to this situation, this paper proposes a hybrid particle swarm optimization and bacterial foraging optimization strategy (PSO-BFO) based on mathematical twin technology, which can signif-icantly improve the lightweight optimization effect of automotive carbon fiber composite battery boxes and achieve an integrated lightweight design of structure, materials and per-formance. However, no relevant methods have been applied to the lightweight design of hy-brid material automotive battery box assemblies.

Therefore, this paper proposes a novel optimization design method for composite battery cases to address the above issues. Based on structural optimization, material substitution and experimental testing, Digital Twinning technology is used in combination with a hybrid par-ticle swarm optimization and bacterial foraging optimization strategy to improve the global optimization capability and convergence speed of composite material battery cases in the lightweight design process. A real-time simulation and monitoring system is used to provide more physical feedback during the PSO-BFO optimization process provides more physical feedback while jointly optimizing key indicators such as strength, stiffness, and manufac-turing cost under multi-objective comprehensive constraints, ensuring that in the pursuit of Lightweight, structural safety and material performance are not compromised, and that an integrated lightweight design of the structure, materials, and performance of the carbon fiber hybrid material battery box can be achieved. This provides an entirely new solution for ad-vanced lightweight design with broad application prospects.

2- The methodology lacks comparisons with existing optimization algorithms, making it difficult to assess the PSO-BFO algorithm's effectiveness.

In order to verify the effectiveness of the hybrid optimization algorithm proposed in this paper, under the same conditions, a single optimization algorithm and a hybrid optimization algorithm are used to optimize the ply design of the lower box of the battery box, and the results are compared and analyzed. Table 1 is a comparison of the results of using different optimization algorithms. From the results, it can be seen that different optimization methods can improve the performance of the battery box and reduce its quality to a certain extent, but using a single optimization algorithm is prone to fall into a local optimal solution. In contrast, the hybrid optimization algorithm can produce more perfect results. The proposed PSO-BFO algorithm improves the mass M of the battery box assembly, the first-order modal frequency of the battery box, the stress under bumpy conditions, and the displacement under bumpy conditions by 40.63%, 33.34%, 83.60%, and 62.80%, respectively. The PSO-GA algorithm improves the mass M of the battery box assembly, the first-order modal frequency of the battery box, the stress under bumpy conditions, and the displacement under bumpy conditions by 33.50%, 29.31%, 83.04%, and 57.87%, respectively. Although both the proposed PSO-BFO algorithm and PSO-GA algorithm can obtain the global optimal solution of complex optimization problems, the results obtained by the PSO-BFO algorithm are more balanced than those of the PSO-GA algorithm. Therefore, the PSO-BFO algorithm proposed in this paper can effectively solve the multi-objective optimization problem of the ply layup sequence of the lower box of the carbon fiber composite battery box.

**Table 1. Comparison of results using different optimization algorithms**

| Optimization methods | Optimization Technology | Optimize results | Improvement rate |
| --- | --- | --- | --- |
| Single optimization algorithm | PSO | Mass = 62.14kg  Modal frequency =45.21Hz  Bump stress =23.245 Mpa  Bump displacement =0.652mm | Mass =29.85  Modal frequency =30.13%  Bump stress =81.69%  Bump displacement =56.3% |
|  | BFO | Mass =60.47 kg  Modal frequency = 43.21Hz  Bump stress =22.842Mpa  Bump displacement =0.648mm | Mass =31.73%  Modal frequency =26.89%  Bump stress =82.00%  Bump displacement =46.67% |
| Hybrid Optimization Algorithm | PSO-BFO | Mass =52.82 kg  Modal frequency = 47.39 Hz  Bump stress =20.821Mpa  Bump displacement =0.452mm | Mass =40.63%  Modal frequency =33.34%  Bump stress =83.60%  Bump displacement =62.80% |
|  | PSO-GA | Mass =58.92 kg  Modal frequency = 44.69 Hz  Bump stress =21.524 Mpa  Bump displacement =0.512mm | Mass =33.50%  Modal frequency =29.31%  Bump stress =83.04%  Bump displacement =57.87% |

3- The finite element modeling section should include a mesh sensitivity analysis for validation.

According to the relevant national standard GB/T 33582-2017 “General Rules for Finite Element Mechanical Analysis of Mechanical Product Structures”, the mesh quality of the finite element model of the battery box assembly established in this paper was checked, and the results are shown in Table 1. It can be seen from Table 2 that the mesh quality of the battery box finite element model established in this paper meets the quality standards specified by the regulations. Therefore, the established battery box assembly finite element model can accurately model the actual situation of the battery box assembly.

Table 2. Mesh quality evaluation criteria

|  | **Mesh size** | **Aspect ratio** | **Warpage** | **Skew** | **Jacobin** | **Angle quad** | **Angle tria** | **percentage** |
| --- | --- | --- | --- | --- | --- | --- | --- | --- |
| **Regulatory standards** | **[5,15]** | **≤ 5** | **≤ 15** | **≤ 40°** | **≥ 0.6** | **[40°, 140°]** | **[30°, 120]** | **≤ 5%** |
| **This model** | **5** | **3.2** | **11** | **30** | **0.7** | **[70°, 120°]** | **[50°, 100°]** | **0.26** |

4- Sources of the 3.67% error in modal frequencies are not discussed and should be clarified.

Thank you very much for your suggestion. We are deeply sorry for our carelessness. We have made additional explanations in the original text, and the additional explanations have been marked in red text.

The maximum error between the simulated modal frequency and the experimental modal frequency is 3.67%. The reason for this may be that there is a deviation between the true coordinate angle of the triaxial accelerometer and the modeling angle, which leads to the maximum error of 3.67% between the simulated modal frequency and the experimental modal frequency. We have already made additional explanations in the article.

5- Figures and tables need annotations to highlight key observations clearly.

Dear reviewer, thank you for your valuable comments! We have optimized the chart captions in the paper to highlight the key observations more clearly. For each chart, we have added more detailed descriptions to more intuitively present the trends and conclusions reflected by the data. In addition, we have further improved the interpretation of key data points so that readers can more easily understand the information conveyed by the charts and strengthen their connection with the core content of the paper. I hope these modifications can improve the readability and accuracy of the paper. Thank you again for your review and suggestions, and look forward to your further feedback. For example, Figures 5, 38, 39, 40, 41, 44, 46, and Table 12. We have provided additional explanations in the manuscript to make it easier to see the key results.

6- The scalability of the proposed method to other automotive components is not addressed.

Regarding the question you raised about applying the method in this article to other automotive parts, other researchers in our research group have successfully applied this method to the front floor and B-pillar of the car, and have carried out physical verification. The relevant research work will be released in the near future.

7- Statistical validation or repeatability of the experimental results is lacking and should be included.

Thank you very much for your suggestion. We are deeply sorry for the inaccurate expression. We have made additional clarifications in the original text, and the additional clarifications have been marked in red text in the manuscript.During the experiment, we conducted multiple groups of experimental controls for each test to ensure the credibility of the test results.

Among them: In the free modal test, the acquisition of modal vibration signal is completed through multiple point measurements. At the same time, in order to reduce the test error and ensure the test accuracy, 20 groups of tests are carried out each time, and then the pathological samples are removed and the average value is taken as the result of each measurement.

In the process of obtaining carbon fiber composite material parameters, we use 6 groups of repeated tests for each test and take the average value of the test results to ensure the credibility of the test results obtained.

8- The literature review could be expanded to include recent advancements in digital twin technology.

Thank you for your suggestions, which are crucial to enriching our work. We have added the latest articles on the progress of digital twin technology to the text, and the added content has been marked with red text.

9- Intermediate optimization results, such as fitness evolution, are not shown and should be added for better visualization.

After repeated tests, the PSO-BFO hybrid algorithm can effectively avoid falling into the local optimum and accelerate the convergence speed of particles by combining the global and local search capabilities of the BFO algorithm and the PSO algorithm. Its convergence effect is better than that of the BFO algorithm and the PSO algorithm. The convergence effect test is shown in Figure 1.

The algorithm iteration process is shown in Figure 1. The PSO-BFO algorithm has a fast convergence speed and high precision. After reaching convergence, the PSO-BFO curve shows slight fluctuations, indicating that it still has a certain global search capability, which helps to refine the optimal solution. The PSO algorithm converges faster, but slightly slower than the PSO-BFO algorithm. The final fitness value is higher than the PSO-BFO algorithm, indicating that its optimization effect is inferior to the PSO-BFO algorithm, but it still has good global optimization capabilities. The BFO algorithm has the slowest convergence speed and the highest final fitness value, indicating that under the same computing conditions, its search efficiency and accuracy are not as good as the other two algorithms.

**Fig 1. Comparison of Convergence Characteristics of PSO, PSO-BFO, and BFO Algorithms**

10- The structural design section could benefit from discussing the durability of the materials in harsh conditions.

Thank you for your suggestions, which are of great importance to our work. Due to the length of the paper and the limitations of the research, this paper does not study the durability of materials under harsh conditions, so we have added relevant literature in the introduction to understand the research content in this area. We have updated these contents in the introduction, and the additions are marked in red text. Thank you very much for your valuable comments and suggestions, which have played a positive role in promoting the improvement of our research. If there are further revisions, we are also very happy to make corresponding adjustments.

11- Details on the choice of excitation point in the experimental setup need clarification.

In the battery box modal test, the optimal excitation point is the one that can maximize the excitation force to excite the various modes of the battery box. The selection criteria for the excitation point are as follows:

1. The excitation point should be far away from the nodes or nodal lines of each mode to avoid mode loss due to insufficient energy input. If the excitation point is located at the node of a certain mode, the mode will not be effectively excited.

2. Choose a place with high rigidity and convenient for excitation. In addition, it is also necessary to consider whether the exciter is easy to install at the selected excitation point. Otherwise, it will not be able to effectively excite and other candidate points need to be re-evaluated. Therefore, the middle edge of the bottom of the battery box is selected as the excitation point in this test.

**Reviewer #2:**

**Dear reviewer:**

**We are very grateful for your professional review of our article. As you are concerned, there are several issues that need to be addressed. According to your suggestions, we have made a lot of revisions to the original manuscript, and the detailed revisions are as follows:**

1- The paper presents improvement rates in battery box performance metrics, but no confidence intervals, statistical significance tests, or sensitivity analysis are provided. Given the computational nature of the study, variance in optimization outcomes should be reported.

Dear reviewer, thank you for your review and valuable comments on our paper. Our research is based on the optimization design of the test and simulation model of the original battery box, so the credibility of the optimization results is mainly supported by the consistency of simulation and test data. Before optimization, the maximum error rate between the free modal test frequency and the simulated modal frequency of the original battery box was 3.67%, indicating that the simulation model reflects the actual situation well. The optimized battery box uses SMC upper cover, and the maximum error between its free modal simulation analysis and modal test frequency is 3.15%, which further proves the reliability of the simulation method. Therefore, although the confidence interval is not provided in the study, the high consistency between simulation and experiment can serve as a support for the reliability of the optimization results.

Given the computational nature of the study, the change in the modal frequency of the battery box upper cover before and after optimization can intuitively reflect the effectiveness of the optimization design. The first-order modal frequency increased from 20.98 Hz to 35.82 Hz, and the performance was improved by 70.73%. The comparison results before and after optimization show that the stiffness of the battery box upper cover has been significantly enhanced, and the optimization scheme has practical value in engineering applications.

Regarding sensitivity analysis, the reliability of the optimization scheme mainly depends on the high consistency between simulation and test data, which is verified by the significant improvement of modal frequency before and after optimization. Since the optimization design is based on a simulation model that has been verified by experiments, and the optimization results show obvious performance improvement, the optimization trend and improvement are sufficient to prove the effectiveness of the optimization design, and the study did not conduct further sensitivity analysis. Thank you for your review and valuable suggestions, we look forward to your further feedback!

2- The study primarily relies on finite element simulations to evaluate the optimized battery box's structural behavior. However, experimental validation is missing. The absence of load tests, vibration tests, or impact assessments makes it difficult to confirm the claimed improvements.

Thank you very much for your suggestions, which are of vital importance to our work. We have included the relevant trials in the article, and the additions are marked in red text. When we submitted the manuscript, the finished SMC composite material upper cover was still in production. Now we have completed the production of the finished SMC composite material upper cover. We supplemented the free modal test of the battery box SMC upper cover and compared it with the experimental modal frequency to verify the correctness of its structure. Due to time constraints, the finished carbon fiber composite lower box has not yet been completed. We will supplement this aspect of the test in future work to enrich our research results. Thank you again for your suggestions.

The free modal test of the SMC composite upper cover can verify the reliability of the established upper cover finite element model. In this test, rubber ropes are used to connect the SMC composite upper cover to simulate the free boundary of the battery box upper cover. Four hydraulic cranes are used to control the hanging height of the battery box upper cover to keep the battery box upper cover in a horizontal state during the test. The specific hanging method is shown in Figure 1. In this free modal test of the battery box upper cover, a force hammer is used as the excitation device. The signals of each sensor are collected through the signal acquisition software. The modal test natural frequency of the SMC composite material upper cover is obtained through analysis and processing. Several groups of experimental controls were carried out for this test to ensure the credibility of the free modal test results.The free modal test process of the SMC composite material upper cover is shown in Figure 2.

**(a)	(b)**

**(c)	(d)**

**Fig 2. Free modal test of SMC upper cover.** (a) Connection of test instrument (b) Test hammer equipment (c) Support method of upper cover (d) Selection of sampling points

The comparison between the free modal test and finite element simulation of the SMC composite upper cover is shown in Table 3. Among them, the maximum error between the free modal test frequency and the simulated modal frequency is 3.15%. The possible reason is that there is a deviation between the real coordinate angle of the three-axis acceleration sensor and the modeling angle, which leads to a maximum error of 3.15% between the simulated modal frequency and the free modal test modal frequency, but meets the accuracy requirements. It can be seen that the established finite element model can better replace the actual battery box cover for modal analysis research, which further verifies the accuracy of the finite element model and the feasibility of the analysis method used.

**Table 3. Comparison of simulation and test results of the first four modal frequencies of the upper cover**

| **Frequency Order** | **Simulation frequency/Hz** | **Test frequency/Hz** | **Error %** |
| --- | --- | --- | --- |
| **1** | **35.82** | **34.98** | **2.40** |
| **2** | **59.71** | **58.14** | **2.70** |
| **3** | **66.14** | **64.12** | **3.15** |
| **4** | **85.30** | **83.46** | **2.20** |

3- The influence of different fiber orientations in the composite layup is not systematically investigated. The PSO-BFO hybrid algorithm optimizes layup sequences, but the paper does not analyze how small deviations in fiber angles (±5° or ±10°) affect stress distribution, stiffness, or failure modes.

Thank you very much for your suggestions. Your suggestions have a vital impact on our work. This issue is very professional, which involves key scientific issues in the design of carbon fiber composite material layup. The reason why we did not study the effect of different fiber orientations is that our paper is based on design optimization at the engineering application level. We actually made an assumption. We combined it with the actual engineering requirements. The actual engineering requirements are to design the carbon fiber composite material layup according to the layup angles of 0°,+45°,-45°,90°. This is relatively regular. We optimize the design of carbon fiber composite materials according to the most commonly used layup method in the enterprise, so we set the layup method to be like this. This layup method assumes that there is no slight deviation in the fiber angle. Of course, it is very important to study the effect of slight deviation in the fiber angle on stress distribution, stiffness or failure mode, but this article does not involve scientific issues here. We only did engineering applications. However, we will strengthen this research in subsequent studies to enrich our research content.

4- The crashworthiness of the optimized battery box is not discussed. Since the battery box is a critical safety component, it should be evaluated under impact scenarios in addition to static and modal analyses.

Thank you very much for your suggestion, which is of vital importance to our work. We have added relevant simulations, and the added content has been marked with red text. Regarding the dynamic performance of the battery box, we selected random vibration conditions and battery box extrusion conditions to evaluate the dynamic performance of the battery box.

Random vibration conditions

The optimized model is used to perform performance analysis and verification of random vibration conditions of the battery box according to relevant standards to ensure that the product meets the use requirements and further verify the feasibility of the optimization method. The vibration excitation that the battery box is subjected to mainly comes from the vibration caused by uneven road surface, which is transmitted through the connection between the vehicle and the battery box. Due to the uncertainty of road conditions, the excitation of the battery box is also random. Therefore, the study of random vibration is of great significance for the performance evaluation of the battery box assembly. Through random vibration analysis, the structural response can be effectively evaluated and the fatigue life can be predicted. According to the GB 38031-2020 regulatory standard, a 21-hour vibration test will be carried out in each axis during the test to observe whether there are abnormal phenomena such as leakage, fire or damage. If the above occurs, the battery box is deemed to be non-compliant with the standard requirements. This article focuses on the rupture of the box during the test. The power spectral density (PSD) data decomposition of each axis is detailed in the table 4-6 below.

**Table 4. X-axis PSD values**

| **Frequency (Hz)** | | **PSD(g^2^/Hz)** | **PSD(m/s^2^)^2^/Hz)** |
| --- | --- | --- | --- |
| 5 | 0.0125 | | 1.2 |
| 10 | 0.03 | | 2.89 |
| 20 | 0.03 | | 2.89 |
| 200 | 0.00025 | | 0.02 |
| RMS | 0.96g | | 9.42 m/s^2^ |

**Table 5. Y-axis PSD values**

| **Frequency (Hz)** | **PSD(g^2^/Hz)** | **PSD(m/s^2^)^2^/Hz)** |
| --- | --- | --- |
| 5 | 0.01 | 0.96 |
| 10 | 0.015 | 1.44 |
| 20 | 0.015 | 1.44 |
| 50 | 0.01 | 0.96 |
| 200 | 0.0004 | 0.04 |
| RMS | 0.95g | 9.32 m/s^2^ |

**Table 6. Z-axis PSD values**

| **Frequency (Hz)** | **PSD(g^2^/Hz)** | **PSD(m/s^2^)^2^/Hz)** |
| --- | --- | --- |
| 5 | 0.05 | 4.81 |
| 10 | 0.06 | 5.77 |
| 20 | 0.06 | 5.77 |
| 200 | 0.0008 | 0.08 |
| RMS | 1.44g | 14.13 m/s^2^ |

The random vibration analysis results in each direction are shown in Figures3, 4and 5. As can be seen from the figure, the random vibration loading point response curves in the X, Y and Z directions are consistent with the national standard input loading curve, indicating that the random vibration simulation analysis results are reliable and the model has good accuracy.

From the RMS stress cloud diagrams of Figure xx and Figure xx, it can be seen that the maximum RMS stress values in the X and Y directions are 1.24Mpa and 0.28Mpa respectively, which appear around the upper right corner of the connection between the lower box and the side wall, which is much smaller than the yield limit of each material, and the overall stress is at a low level.

From the RMS stress cloud diagram in Figure xx, it can be seen that the maximum RMS stress value in the Z direction is 24.56MPa, which occurs around the connection between the lifting lug and the side panel, and is much smaller than the yield limit of each material. The overall stress is higher than the RMS stress values in the X and Y directions, and lower than the yield limit of each material. The overall stress is at a relatively low level.

(a)	(b)

**Fig 3. X-direction random vibration. (a) Loading point response curve (b) RMS stress cloud diagram.**

(a)	(b)

**Fig 4.** **Y-direction random vibration. (a) Loading point response curve (b) RMS stress cloud diagram**

(a)	(b)

**Fig 5.** **Z-direction random vibration. (a) Loading point response curve (b) RMS stress cloud diagram.**

Battery box extrusion simulation

The battery box is a key component of new energy vehicles. Its extrusion may cause damage to the internal battery cells, circuit control systems, and high and low voltage wiring harnesses, which may affect the power system at best and cause fire or explosion at worst. During the simulation process, an extrusion table model needs to be established, which consists of two surfaces, as shown in Figure xx. The battery box is placed on the table, and a cylinder is used to apply extrusion along the X-axis and Y-axis directions until the pressure reaches 100KN or the deformation reaches 30% of the box size. The simulation analysis uses LS-DYNA software. First, the finite element model of the battery box is imported into the LS-DYNA module of Hypermesh, all two-dimensional units are set to self-contact, and the pre-built extrusion bench model is loaded, as shown in Figure 6.

（a）	（b）

**Fig 6. Finite element model of extrusion simulation conditions.** (a) Finite element model of extrusion platform (b) Model diagram of extrusion conditions

Create contact between the battery box and the extrusion stand. The stand surface is a fixed part, and it is in fixed surface contact with the battery box; the semi-cylinder is a moving part, and it is in moving surface contact with the battery box, as shown in the following figure 7.

(a)	(b)

**Fig 7. Contact conditions in extrusion simulation.** (a) moving surface contact (b) fixed surface contact.

The extrusion bench is made of rigid material with a thickness of 1mm. The material parameters of the moving parts and fixed parts are set separately, and the fixed parts are fully constrained with six degrees of freedom. The cylindrical moving parts are constrained with six degrees of freedom of rotation and five degrees of freedom of translation, and only X-direction movement is allowed.

This simulation uses forced displacement loading to avoid the initial velocity being reduced due to extrusion release, which affects the working condition requirements. The displacement curve is applied through the boundary forced displacement load, and the displacement is set to 200mm within 20ms. After completing the material properties and load condition settings, the extrusion simulation model in the X-axis direction can be established.When modeling in the Y-axis direction, the battery box needs to be rotated 90° along the Z axis and placed on the test bench. The cylindrical moving part still moves in the X-axis direction without adjusting the direction. After completing the extrusion modeling in the X and Y directions, the model is imported into LS-DYNA for analysis.

According to regulatory requirements, when the extrusion force reaches 200kN, the battery cells, circuit control system and high and low voltage harnesses inside the battery box must remain intact. When simulating the extrusion condition, a uniform forced displacement is applied to the moving part within 0.02 seconds to obtain the extrusion force-displacement curve. Simulations are performed in the X-axis and Y-axis directions respectively, and the extrusion condition in the X-axis direction is analyzed first. The extrusion analysis results are obtained using LS-DYNA, and the extrusion force-time and extrusion force-displacement curves are displayed in Hyperview. The results are shown in Figure 8.

(a)	(b)

**Fig 8. Extrusion curve in the X-axis direction.** (a) Forced displacement-stress diagram (b) Time-stress relationship diagram.

According to the curve data, when the extrusion force reaches 200kN, the displacement of the cylinder is 50mm, and the corresponding extrusion time is 0.01ms. Import the LS-DYNA simulation results into Hyperview, and extract the stress and displacement cloud diagrams. The extrusion and intrusion of the battery box at 0.01ms can be clearly observed, as shown in Figure 9. The maximum deformation of the battery pack structure at the moment when the extrusion force in the X direction reaches 200kN is 10.60mm, and the position appears at the connection between the upper and lower boxes at the front end. The distance between the connection and the battery module is about 100mm, and the deformation is much smaller than this value, so the battery module will not be squeezed.

（a）	（b）

**Fig 9. Extrusion cloud map in the x-axis direction.** (a) displacement cloud map (b) stress cloud map.

Then, the extrusion simulation condition of the battery box in the Y-axis direction is analyzed. The extrusion condition analysis results in LS-Dyna, the extrusion force and action time, and the extrusion force and displacement curves are displayed in Hyperview, and the analysis results can be obtained as shown in Figures 10.

(a)	(b)

**Fig 10. y-axis extrusion curve**. (a) forced displacement-stress diagram (b) time-stress relationship diagram.

According to the curve analysis, when the extrusion force reaches 200kN, the dis-placement of the cylinder is 7mm, 10mm, and 76mm, and the corresponding extrusion time is 0.0014ms, 0.002ms, and 0.015ms. Comprehensively comparing the three sets of data, when the displacement reaches 76mm and the action time is 0.015ms, the battery box is most af-fected. By reading the stress and displacement cloud map of the extrusion condition analysis of LS-DYNA through Hyperview, the extrusion intrusion of the battery box at 0.015ms can be clearly observed, as shown in Figure 11.

(a)	(b)

**Fig.11 y-axis extrusion cloud map.** (a) displacement cloud map (b) stress cloud map.

As for the Y direction, the maximum deformation of the battery pack structure when the extrusion force reaches 200kN is 11.05mm, which occurs at the connection between the upper and lower boxes on the side. The minimum distance between the side of the battery pack box and the battery module is 20mm, and the deformation is much smaller than this value, so the battery module will not be squeezed. Based on the extrusion results in the X and Y directions, it can be judged that the carbon fiber composite material used has excellent specific stiffness performance. When the extrusion force reaches 200kN, the deformation of the box structure is at a low level, which meets the standard regulations and usage requirements.

5- The transition between the SMC composite upper panel and the carbon fiber composite lower panel could lead to localized stress concentration. The paper lacks stress contour plots highlighting these transition regions and does not mention how delamination or interface debonding risks were mitigated.

Thank you very much for your suggestion, which is very important for our work. You raised the possibility that the transition between the SMC composite upper cover and the carbon fiber composite lower box may lead to local stress concentration, which is a very good question. There is a problem of local stress concentration in the transition area between the SMC composite upper cover and the carbon fiber composite lower box. We use bolts to connect the SMC composite upper cover and the carbon fiber composite battery box lower box. Taking the bumping condition as an example, the stress concentration occurs in the transition area between the two at the bolt hole. It can be seen from Figure 12 that the stress values in the transition area between the SMC composite upper cover and the carbon fiber composite lower box are within the normal range. We believe that this risk is controllable. As you said, if the SMC composite upper cover and the carbon fiber composite battery box lower box are glued, there will be a risk of delamination and interface debonding. However, in order to avoid this situation, we use bolt connection in this article. In the finite element simulation, we use RBE2 rigid unit simulation for bolt connection to avoid the risk of delamination and interface debonding. Thank you again for your suggestion.

(a)	(b)

**Fig 12. Stress cloud diagram of upper cover and lower box under bumpy conditions.** (a) Battery box cover (b)Battery box lower body

6- The study does not compare the optimized hybrid composite battery box with other alternative lightweight designs to contextualize the improvements.

Thank you very much for your suggestions, which are of vital importance to our work. Our battery box is made of high-strength steel, which has replaced ordinary steel materials. The SMC composite upper cover we proposed in this article and the lower box material are made of carbon fiber composite material. This design is lighter than high-strength steel. In terms of material selection, we have manually selected materials to make the battery box as light as possible. But it is inevitable that this design will lead to a huge increase in costs, which we have explained in the article. In addition to comparing the materials and costs, we also compared the optimization algorithms. We applied the PSO-BFO algorithm to the carbon fiber composite material layup design, and we also compared this hybrid algorithm with other algorithms. Such as table 7, so that readers can better understand the improvement situation

**Table 7. Comparison of results using different optimization algorithms**

| **Optimization methods** | **Optimization Technology** | **Optimize results** | **Improvement rate** |
| --- | --- | --- | --- |
| Single optimization algorithm | PSO | Mass = 62.14kg  Modal frequency =45.21Hz  Bump stress =23.245 Mpa  Bump displacement =0.652mm | Mass =29.85  Modal frequency =30.13%  Bump stress =81.69%  Bump displacement =56.3% |
|  | BFO | Mass =60.47 kg  Modal frequency = 43.21Hz  Bump stress =22.842Mpa  Bump displacement =0.648mm | Mass =31.73%  Modal frequency =26.89%  Bump stress =82.00%  Bump displacement =46.67% |
| Hybrid Optimization Algorithm | PSO-BFO | Mass =52.82 kg  Modal frequency = 47.39 Hz  Bump stress =20.821Mpa  Bump displacement =0.452mm | Mass =40.63%  Modal frequency =33.34%  Bump stress =83.60%  Bump displacement =62.80% |
|  | PSO-GA | Mass =58.92 kg  Modal frequency = 44.69 Hz  Bump stress =21.524 Mpa  Bump displacement =0.512mm | Mass =33.50%  Modal frequency =29.31%  Bump stress =83.04%  Bump displacement =57.87% |

7- The paper does not include a convergence plot for the hybrid PSO-BFO algorithm. Given that hybrid swarm-based optimizers may experience premature convergence, it is essential to demonstrate whether the objective function stabilizes and how many iterations were required.

Thank you for your suggestion, which is of vital importance to our work. We are deeply sorry for our negligence. We have added the convergence diagram of the PSO-BFO algorithm to achieve better visualization. We have added this content in the article, and the added content has been marked with red text.

After repeated tests, the PSO-BFO hybrid algorithm can effectively avoid falling into the local optimum and accelerate the convergence speed of particles by combining the global and local search capabilities of the BFO algorithm and the PSO algorithm. Its convergence effect is better than that of the BFO algorithm and the PSO algorithm. The convergence effect test is shown in Figure 13.

The algorithm iteration process is shown in Figure xx. The PSO-BFO algorithm converges quickly and has high accuracy. It reaches convergence after 6500 iterations. The PSO-BFO curve shows slight fluctuations, indicating that it still has a certain global search capability, which helps to refine the optimal solution. The PSO algorithm converges quickly, but slightly slower than the PSO-BFO algorithm. The final fitness value is higher than the PSO-BFO algorithm, indicating that its optimization effect is inferior to the PSO-BFO algorithm, but it still has good global optimization capabilities. The BFO algorithm converges the slowest, and the BFO algorithm has the highest final fitness value, indicating that under the same computing conditions, its search efficiency and accuracy are not as good as the other two algorithms.

**Fig 13. Comparison of Convergence Characteristics of PSO, PSO-BFO, and BFO Algorithms.**

8- While the PSO-BFO hybrid approach is novel, the paper does not compare its performance with other optimization algorithms. Benchmarking against existing approaches would strengthen the validity of its effectiveness.

In order to verify the effectiveness of the hybrid optimization algorithm proposed in this paper, under the same conditions, a single optimization algorithm and a hybrid optimization algorithm are used to optimize the ply design of the lower box of the battery box, and the results are compared and analyzed. Table 8 is a comparison of the results of using different optimization algorithms. From the results, it can be seen that different optimization methods can improve the performance of the battery box and reduce its quality to a certain extent, but using a single optimization algorithm is prone to fall into a local optimal solution. In contrast, the hybrid optimization algorithm can produce more perfect results. The proposed PSO-BFO algorithm improves the mass M of the battery box assembly, the first-order modal frequency of the battery box, the stress under bumpy conditions, and the displacement under bumpy conditions by 40.63%, 33.34%, 83.60%, and 62.80%, respectively. The PSO-GA algorithm improves the mass M of the battery box assembly, the first-order modal frequency of the battery box, the stress under bumpy conditions, and the displacement under bumpy conditions by 33.50%, 29.31%, 83.04%, and 57.87%, respectively. Although both the proposed PSO-BFO algorithm and PSO-GA algorithm can obtain the global optimal solution of complex optimization problems, the results obtained by the PSO-BFO algorithm are more balanced than those of the PSO-GA algorithm. Therefore, the PSO-BFO algorithm proposed in this paper can effectively solve the multi-objective optimization problem of the ply layup sequence of the lower box of the carbon fiber composite battery box.

**Table 8. Comparison of results using different optimization algorithms.**

| **Optimization methods** | **Optimization Technology** | **Optimize results** | **Improvement rate** |
| --- | --- | --- | --- |
| Single optimization algorithm | PSO | Mass = 62.14kg  Modal frequency =45.21Hz  Bump stress =23.245 Mpa  Bump displacement =0.652mm | Mass =29.85  Modal frequency =30.13%  Bump stress =81.69%  Bump displacement =56.3% |
|  | BFO | Mass =60.47 kg  Modal frequency = 43.21Hz  Bump stress =22.842Mpa  Bump displacement =0.648mm | Mass =31.73%  Modal frequency =26.89%  Bump stress =82.00%  Bump displacement =46.67% |
| Hybrid Optimization Algorithm | PSO-BFO | Mass =52.82 kg  Modal frequency = 47.39 Hz  Bump stress =20.821Mpa  Bump displacement =0.452mm | Mass =40.63%  Modal frequency =33.34%  Bump stress =83.60%  Bump displacement =62.80% |
|  | PSO-GA | Mass =58.92 kg  Modal frequency = 44.69 Hz  Bump stress =21.524 Mpa  Bump displacement =0.512mm | Mass =33.50%  Modal frequency =29.31%  Bump stress =83.04%  Bump displacement =57.87% |

9- The study does not assess how variations in material properties (e.g., fiber volume fraction, matrix stiffness, interlaminar shear strength) influence the optimization results. Given manufacturing inconsistencies, this should be addressed.

Thanks to the reviewer for his valuable comments. Our research focuses on the optimization method of carbon fiber reinforced composite (CFRP) layup design, mainly focusing on how to improve the efficiency of the optimization algorithm and the performance of the optimization scheme. Therefore, in this study, we assume that the material properties (such as fiber volume fraction, matrix stiffness, and interlaminar shear strength) are fixed values to simplify the optimization calculation and focus on the study of layup optimization strategy.

We understand that there may be some fluctuations in material properties during the manufacturing process, but this problem falls into the category of material processing and manufacturing variation, which is different from the core optimization method of this study. Therefore, we did not conduct an in-depth discussion on the impact of material property changes in this paper. We believe that the method proposed in this study can still provide valuable ideas for composite structure optimization, and future research can be further expanded on this basis to cover manufacturing errors and material uncertainty factors. Thank you for your valuable suggestions.

10- The shear properties of the optimized composite layup are not analyzed. Battery boxes may be subjected to out-of-plane shear loads, which could lead to delamination. A short-beam shear test or interlaminar tension test should be conducted.

Dear reviewer, thank you for your valuable comments. Your suggestions are of great value to the improvement of the research. You pointed out that the optimized composite layup may be subjected to out-of-plane shear loads, resulting in delamination, and suggested that short beam shear tests or interlaminar tensile tests be performed to analyze its interlaminar shear performance.

In this study, we mainly used tensile tests and three-point bending tests to evaluate the mechanical properties of the optimized composite material. The experimental diagram is shown in Figure 14, and the following experimental data were obtained:

The tensile data of the carbon fiber spline are shown in Table 9. The average tensile strain at break of the spline is 1.22%, the average tensile modulus is 66.60 GPa, and the average tensile strength is 778.86 MPa.

**Table 9. Tensile test results.**

| **Sample** | **Tensile strain at break/%** | **Tensile elastic modulus/MPa** | **Tensile strength /MPa** |
| --- | --- | --- | --- |
| 1 | 1.12 | 65219.71 | 691.98 |
| 2 | 1.41 | 61563.11 | 911.43 |
| 3 | 1.23 | 76494.46 | 690.87 |
| 4 | 1.16 | 63702.69 | 799.53 |
| 5 | 1.17 | 66040.83 | 800.50 |

Figure 14(d) is the stress-strain curve of the specimen bending test. From the test results, it can be found that the average bending modulus of the composite material specimen is 61.31 GPa, the average bending strength is 541.55 MPa, and the average strain at interlaminar failure is 0.83%.

(a)	(b)

(c)	(d)

**Fig 14. test diagram.** (a) Tensile specimen with reinforcement sheet (b) Tensile fracture surface (c) Three-point bending fracture micromorphology (d) Three-point bending test stress-strain curve.

According to the results of the three-point bending test, the interlaminar failure of the specimen occurred at a strain of 0.83%, while the average tensile strain at break in the tensile test was 1.22%, indicating that the deformation capacity of the optimized composite material under bending loading is close to that of the tensile condition, and that good interlaminar bonding performance is maintained under higher loads. Generally, if the interlaminar shear performance of the composite material is poor, early interlaminar delamination failure is likely to occur near the fulcrum (shear stress-dominated area) in the bending test. However, in the three-point bending test of this study, we did not observe obvious interlaminar delamination failure, and the specimens mainly showed overall bending failure or compression yield, which indicates that the optimized laminate structure has good interlaminar bonding strength.

In addition, the interlaminar failure strain in the bending test is lower than the tensile fracture strain, which further indicates that the material does not fail prematurely under the load condition dominated by interlaminar shear. Although the short beam shear test and interlaminar tensile test can measure the interlaminar shear strength more directly, we did not find an obvious interlaminar delamination trend from the existing experimental data, indicating that the optimized laminate structure still has good interlaminar stability.

We fully recognize the importance of short beam shear test and interlaminar tensile test in directly measuring interlaminar shear performance. However, due to the limitation of experimental equipment, we are currently unable to conduct these tests. Therefore, we used three-point bending test in this study, combined with experimental phenomenon analysis, to indirectly evaluate the interlaminar bonding performance of the optimized laminate. We plan to supplement the short beam shear test or interlaminar tensile test in future research to further verify the interlaminar shear performance of the optimized composite material.

Thank you again for your review and valuable suggestions. I hope these supplementary analyses can meet your requirements and look forward to your further feedback!

11- The paper does not discuss the practical challenges in manufacturing the hybrid composite battery box. Issues like tooling cost, layup reproducibility, curing cycle optimization, and adhesive bonding techniques should be addressed.

Thank you very much for your suggestions, which are very important for our work. The tool cost of this study is relatively high, and the repeatability of the layers is limited by the process of 0°,+45°,-45°,90° layers. Due to the current limitations of the research group, we have not done research on the optimization of the curing cycle and adhesive bonding technology. We are well aware that this is a very important scientific issue, and this is the future research direction of our research group. We will study this aspect in future research. Thank you again for your suggestions. Thank you very much.

12- Given the scope and content of this paper, it may benefit from considering the following related works:

https://doi.org/10.1007/s00521-024-09494-4

<https://doi.org/10.1016/B978-0-443-13191-2.00015-8>

Thank you very much for your suggestions, which are of vital importance to our work. After reading the article, we all agree that the literature is highly relevant to our research. We have cited these articles in the revised manuscript, and the additional content has been marked in red text. These literature materials are of great significance to our improvement and enrichment of the content of this article. Thank you again for helping us improve the integrity of the paper. Thank you very much. If you have any further suggestions for revision, we will be happy to make corresponding adjustments.
